# Giant Magnetoresistance Sensors: A Review on Structures and Non-Destructive Eddy Current Testing Applications

**DOI:** 10.3390/s16030298

**Published:** 2016-02-26

**Authors:** Damhuji Rifai, Ahmed N. Abdalla, Kharudin Ali, Ramdan Razali

**Affiliations:** 1 Faculty of Engineering Technology, Universiti Malaysia Pahang, Gambang, Pahang 26300, Malaysia; ahmed@ump.edu.my (A.N.A.); ramdan@ump.edu.my (R.R.); 2 Faculty of Electrical & Automation Engineering Technology. TATI University College, Kemaman 26000, Terengganu, Malaysia; kharudin@tatiuc.edu.my

**Keywords:** giant magnetoresistance, eddy current testing, non-destructive testing

## Abstract

Non-destructive eddy current testing (ECT) is widely used to examine structural defects in ferromagnetic pipe in the oil and gas industry. Implementation of giant magnetoresistance (GMR) sensors as magnetic field sensors to detect the changes of magnetic field continuity have increased the sensitivity of eddy current techniques in detecting the material defect profile. However, not many researchers have described in detail the structure and issues of GMR sensors and their application in eddy current techniques for nondestructive testing. This paper will describe the implementation of GMR sensors in non-destructive testing eddy current testing. The first part of this paper will describe the structure and principles of GMR sensors. The second part outlines the principles and types of eddy current testing probe that have been studied and developed by previous researchers. The influence of various parameters on the GMR measurement and a factor affecting in eddy current testing will be described in detail in the third part of this paper. Finally, this paper will discuss the limitations of coil probe and compensation techniques that researchers have applied in eddy current testing probes. A comprehensive review of previous studies on the application of GMR sensors in non-destructive eddy current testing also be given at the end of this paper.

## 1. Introduction

Non-destructive testing (NDT) is a quality control tool that is extremely important in heavy engineering sectors such as the petroleum and gas industry. It is the last test before a component, system or process are deemed safe to run. In the petroleum industry, non-destructive testing is widely used in detecting defects in the storage tanks and pipes that deliver oil and gas. Failure to detect and provide accurate information about the status of quality components, systems or processes may result in an accident that ends with the destruction of property and loss of life. Table 1 shows an overview of the major nondestructive testing techniques that are widely used in the oil and gas industry. With of the existing conventional NDT it is impossible to conduct inspections through the hundreds kilometers of a pipeline system used in the oil and gas industry. Thus, a simple and quick method to inspect the defects in large pipe systems is necessary.

Eddy current testing (ECT) techniques are a non-destructive evaluation method for defect inspection on conductive materiald. The main advantages of ECT are that a diversity of inspections can be done using this technique, including surface and subsurface material defect inspections, thickness and coating measurements, electrical conductivity measurements for material classification and conductive monitoring during material heat treatment [1]. Compared with another nondestructive testing methods, this technique offers many advantages such as high sensitivity to small defects for tests that require fast scanning inspection. In addition, this technique also known as a technique that requires no physical contact between the probe and test subject [2]. This makes this technique particularly suitable for continuous monitoring of growth defects in pipeline systems.

The sensitivity of non-destructive eddy current testing techniques can be improved by selecting an appropriate probe and an optimum frequency exciting coil for each type of crack and tested material [3,4]. To monitor the growth of cracks in large system structures, the test probe is placed on the detected cracks and the data continuously collected for defect profile analysis [1]. In this application, ECT techniques are more suitable than another non-destructive testing methods. Although ultrasonic testing techniques can provide an accurate defect profile, this technique requires a couplant as a signal transmission medium between the probe and testing material. For this reason, eddy current testing is still considered the best non-destructive testing technique [5]. Table 1 shows a comparison of non-destructive testing methods widely used in industry.

Eddy current testing can also be used to measure material thinning caused by corrosion. This application is widely used to measure the thinning that occurs in aircraft bodies and heat exchanger tubes in nuclear plants.

The novel state of the art non-destructive testing eddy current techniques are derived from electromagnetic principles. The techniques are based on a non-uniform magnetic field induced in the conductive material due to the presence of defects to determine the profile of a defect in the material being inspected [6,7,8]. Various material profiles can be obtained by using different exciting current frequencies. The magnetic field changes may provide information related to any defects inherent in the material, electrical conductivity and permeability of the material after a heat treatment process, surface and subsurface defects. This discovery is the starting point for research on non-destructive testing using the eddy current principle.

The accuracy and effectiveness of the non-destructive eddy current testing technique depend on the sensitivities of the receiving coil probe used to detect the magnetic field changes caused by defects in the material under testing. Application of the giant magnetoresistance (GMR) sensor as a signal receiver has increased the sensitivity of this technique in the detection of small defects [9]. Integrating this system with smart algorithms allows this technique to produce complete and comprehensive profile data. Implementation of GMR sensors in non-destructive eddy current techniques is still new, and many issues related to the hybrid system (coil-GMR) have not been explored by researchers. This paper will describe the implementation of GMR sensorw in non-destructive eddy current testing techniques, a previous study of the applications of GMR sensor in non-destructive eddy current testing and the issues that need to be addressed by future researchers.

## 2. Overview of Giant Magnetoresistance Sensors

Research in the field of magnetic films has been carried out by researchers in recent years. The discovery of giant magnetoresistance (GMR) in 1988 provided scientists with a new perspective for understanding polarized carriers in ferromagnetic metals and possibilities to apply this in new technologies, particularly in non-destructive testing [8,9]. Obeid [10] in his study discussed the details of GMR structures.

GMR sensor have attracted the interest of many researchers and are widely used in various applications because these sensors have high bandwidths and sensitivity independent of the magnetic field. In addition, other advantages are the small dimensions of GMR sensors and the fact they require only a low power source. GMR sensors detect magnetic field vectors along the axis of the sensing track [11].

GMR sensors have higher sensitivity and higher output signal compared to other MR sensors. GMR sensors were introduced in 1936 based on the Mott model [12]. The resistance of the ferromagnetic material in the GMR sensor shows a significant increase when heated above the Curie temperature. Mott investigated the conduction of the GMR sensor by modeling two different channels which have spin up and spin down electrons. He found that the rate of electron scattering in the metal is smaller compared to ferromagnetic materials. This principle is the basis for the development and application of the GMR sensor.

**Figure 1:**
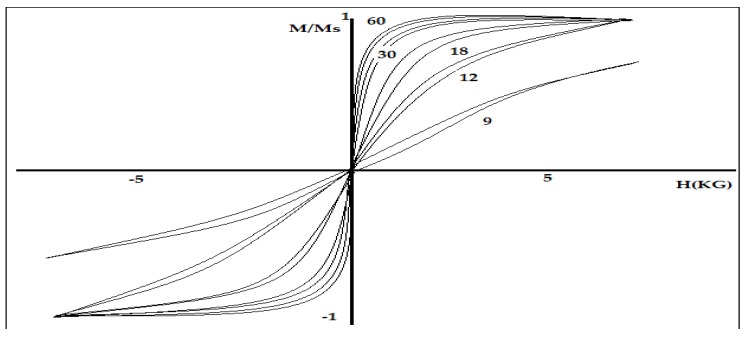
Hysteresis loops for several Fe/Cr for different thickness of Cr and with the presence of magnetic field by Baibich *et al.* [13].

The first principle of the GMR sensor was discovered in 1988 by Baibich *et al.* [13]. They studied the magnetic resistance of the structure layer between the (001)Fe/(001)Cr. The results showed the resistance of the material layer structure depends on the direction of electron scattering rotation between the layers of Fe and Cr. Figure 1 shows the hysteresis loop for Fe /Cr at a temperature of 4.2 K when a magnetic field is applied to the surface of the structure. Their studies showed that antiferromagnetic properties between layers of Fe and Cr increase drastically when the thickness of the Cr is less than 30Å to 9Å.

**Figure 2:**
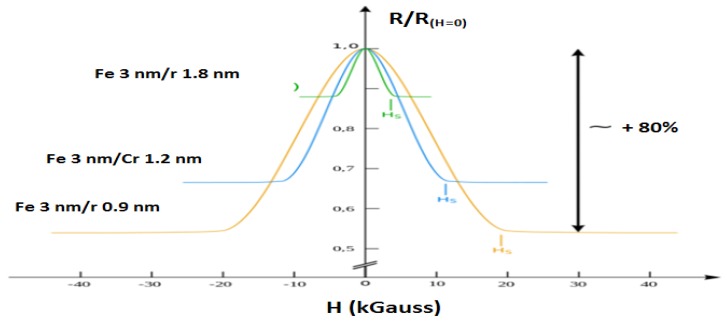
Magnetoresistance of three Fe/Cr superlattices at 4.2 K with different thickness by Baibich *et al.* [13].

Baibich *et al.* [13] found that there was a relationship between the thickness of the Cr layer and the temperature. They found that the magnetic resistance decreases as the thickness increases and the properties of antiferromagnetic Cr(AF) in Fe/Cr structure layer is weak, as shown in Figure 2. With the increase of temperature from 4.2 K to room temperature, magnetic resistance and Hs decreased, where Hs is the magnetic field required to overcome the antiferromagnetic properties in the structural layer of Fe/Cr.

Two types of GMR sensor will be discussed in this paper, the first one is the GMR spin valve and the second one is the GMR multilayer sensor.

### 2.1. Giant Magnetoresistance Spin Valve Sensor

The term "spin valve magnetoresistance" was introduced by Dieny *et al.* [14]. They introduced a GMR sensor structure consisting of two ferromagnetic layers separated by a thin layer of non-ferromagnetic material (NM). The ferromagnetic (FM) layer is a permanent magnetization and soft layer. The soft layer structure has an electron orientation that is easily manipulated by applying an external magnetic field. The orientation of electrons in the ferromagnetic material can be changed from parallel and non-parallel or *vice versa* by change the coercive field in the ferromagnetic layer. Thus, the resistance between these layers depends on the orientation of electron carriers in the layer of the material structure. In the 1950s, the exchange of anisotropy that is caused by the direct exchange of electrons between a ferromagnetic and antiferromagnetic material layer in the GMR structure was studied by Dieny *et al.* [14].

Prinz [15] described the physical origin of the spin valve magnetoresistance by using two principles. He explained that the principles of the GMR sensor are due to the different numbers of spin up and spin down electrons in the conduction band of the GMR sensor material structure. This phenomenon occurs because of an imbalance of electrons on the Fermi energy level and scattering of the spin electrons caused by collisions with impurities in the GMR material structure.

The spin valve principle is based on the orientation of electrons. When the ferromagnetic layers have the same magnetization direction, only one type of electrons (spin down) scatter strongly, which makes a low resistivity material. Conversely, when the GMR sensor layer magnetization directions are opposite to each other, the two types of electrons (spin up and spin down) are scattered significantly which makes the resistivity of the material high.

**Figure 3:**
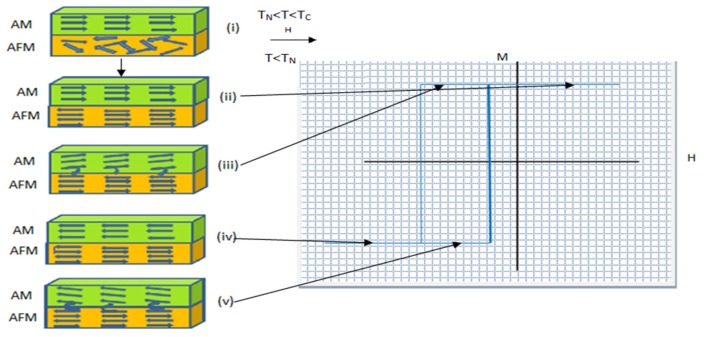
Schematic diagram of the spin valve configuration of FM/AFM (a) at different stages (i) to (v) of an exchange biased hysteresis loop by Nogues *et al.* [16].

Nogues *et al.* [16] explained that anisotropy and exchange bias occur on the surface layer between ferromagnetic and antiferromagnetic (AFM/FM) in the GMR structure. When the magnetic field applied to the GMR structure is in the temperature range between the Neel and Curie temperatures (TN <T <TC), the electron spin in FM materials is aligned with the magnetic field while the electron spin in the AFM remains scattered, as shown in Figure 3(i). When the temperature is reduced to T < TN electron spin in an AFM layer is parallel to the direction of the electron spin in the FM layer. This interaction causes the effective electromagnetic field on the surface of the FM/AFM layer to be zero as shown in Figure 3(ii). If a magnetic field is applied to the GMR structure in opposite directions, the electron spin in the FM layer starts rotating while the electron spin in the AFM layer remained unchanged (Figure 3(iii)).

**Figure 4:**
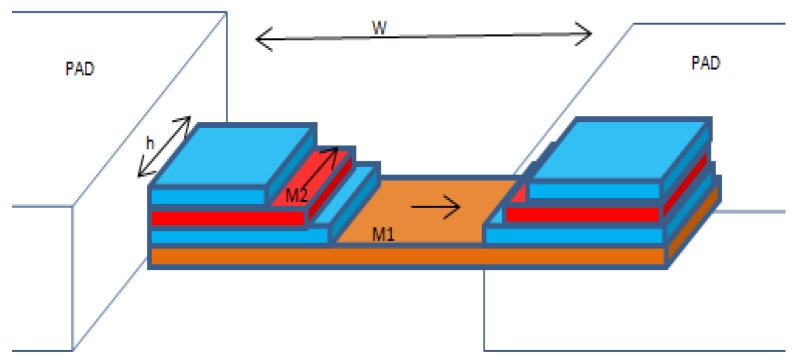
Schematic of a spin valve sensor element. M1 is the free ferromagnetic layer, and M2 is the pinned ferromagnetic layer by Freitas *et al.* [17].

In the mid-1990s, Freitas *et al.* [17] introduced the linear spin valve sensor design as shown in Figure 4. Length and width of the magnet dimensions are *W* and *h*. A material layer of scattered electrons is arranged in a longitudinal orientation. When an excitation magnetic field is applied opposite the spin electrons’ orientation, electron spin in the material will rotate and be parallel to the direction of the applied magnetic field. The scattering angle rotation of electron spins is dependent on the position of electron spins along the surface layer material.

Nolting *et al*. [18] studied the effect arrangement of electron spin structure on the surface multilayer GMR structure. Electron spin in the surface layer has a big impact on the material properties. Direct coupling between the electron spin in the antiferromagnetic layer and the electron spin in ferromagnetic materials becomes very important in the development of magnetic read heads and magnetic memory cells.

There are various types of multi-layer GMR spin valve. The spin valves that most attract the interest of researchers in this field are the top spin valve, the bottom valve and the symmetric spin valve. The differences in this type of spin valve are based on the fabrication process, the material layers used and the structure of the layers.

### 2.2. Giant Magnetoresistance Multilayer Sensor

**Figure 5:**
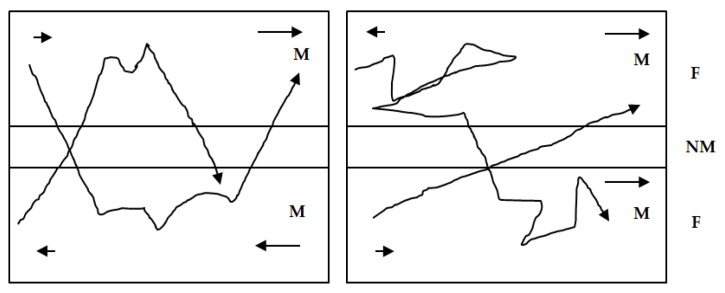
Schematic representation of the basic mechanism of the GMR by Kools [19].

Electron carriers in GMR multilayers sensor structures consisting of two ferromagnetic layers separated by a non-magnetic layer have been discussed by Kools [19]. The ferromagnetic layer has a majority of spin up and spin down electrons moving in scattered motion on this layer. In Figure 5, the scattering of electrons is much stronger if the direction of electron movement is opposite to the direction of the applied external magnetic field. When the magnetic field is parallel, spin down electrons scattered strong and spin up electrons can easily move through the two layers of ferromagnetic and non-ferromagnetic material without scattering. Electron movement is only scattered in one layer when the direction of the external magnetic field applied to the GMR structure is opposite the direction of the electrons.

Lenssen *et al.* [20] discussed the GMR multilayer sensor structure composed of ferromagnetic and nonmagnetic layers. The thickness of the layer structure is selected for electron coupling exchange between the two layers of the material. When an external magnetic field is applied to the GMR structure, electrons in both materials will rotate in the same direction to align in the same configuration. The alignment position of the electron configuration reduces the resistance of the GMR structure.

Several studies have been conducted by researchers to study the behavior of electron movement in two adjacent surface layers of coupling material [21]. The most important thing in a GMR structure is that carrier movement not be locked in the bonds that exists in the ferromagnetic coupling layer to enable the electrons to freely rotate in the direction of the external magnetic field [22].

GMR sensors using the Wheatstone bridge principle were designed and fabricated by Daughton *et al.* [23] in 1994. The objective of this design was to reduce the hysteresis effect and for linear output. The structure of the GMR sensor consists of four resistors. Two resistors are protected from being exposed to a magnetic field and another two unshield active resistors are in between the two flux concentrators. The advantage of this design is that the sensitivity of the GMR sensor can be varied by changing the length and distance between the two flux concentrators.

Rochaz *et al.* [24] introduced a Wheatstone bridge GMR sensor consisting of 21 magnetic layers (NiFe) separated by a nonmagnetic material (Ag). The NiFe thickness they used was 2 nm and the thickness of the Ag spacer was 1.1 nm. The advantage of this GMR structure is its stability when exposed to high temperatures compared with other GMR sensor structure designs that have a Cu layer spacer. In addition, these sensors have a better output linearity and the hysteresis signal is low (less than 1 Oe).

## 3. Types of Non-Destructive Eddy Current Testing Probe

There are mainly two types of eddy current probes: impedance variation probes and excitation-detection probes. Impedance variation probe coils induce an eddy current in the specimen. The secondary flux created by the eddy currents changes the flux coupling the exciting coils and thus the coil impedance. The impedance variation is monitored and measured by instrumentation. In excitation-receive probes, the excitation coils induce eddy currents within the specimen, and the voltage induced in the receive coil by the time varying magnetic field forms the measured signal.

In the past few decades, three different commercial probes have been used widely in the market for the inspection of pipe and tubes: absolute and differential bobbin probes; motorized rotating probe coil (MRPC) with pancake and plus point coils; and array probes, such as X-probe, smart array probe and intelligent probe. Among these probes, the bobbin probe and rotating probe are impedance variation probes, whereas the array probe belongs to a class of excitation-receive probe.

### 3.1. Bobbin Probe

Bobbin probes are reliable and capable of detecting and sizing volumetric defects such as fretting wear and pitting corrosion. The typical scanning speed is up to 1 m/s. Bobbin probes are connected to analog single-frequency instruments with a scope for impedance trajectories display. Bobbin probes have fast scan speeds of approximately 1 m/s and are mainly used for initial detection of possible degradation to determine the areas requiring additional inspection using other probes with improved ability to size and characterize degradation [25]. Two types of bobbin probes are commonly used in tube inspection: absolute bobbin and differential bobbin probes [26].

Absolute bobbin probes operate with single bobbin coil and a second identical reference coil, which is used for electronic balancing and electromagnetically shielded from the inspected tubing. The probe is sensitive to axial cracks in the tube wall. Material property variations, and gradually varying wall thinning, are not detected by differential bobbin probes.

Differential bobbin probes have two coils that are differentially connected. The typical probe outer diameter ranges from 12~30 mm, thickness from 0.7~3 mm and lift-off from 0.8~1.5 mm. The probe operates with the current in one coil 180 degrees out of phase with the current in the other. The recorded signal is the total impedance of the two coils.

Differential bobbin probes are sensitive to small defects and abrupt anomalies such as pitting corrosion and fretting wear, unaffected by lift-off, probe wobble, temperature variations, gradual tube conductivity changes and external interference. The probes are also not sensitive to gradual changes such as metallurgical variations, geometry and slowly increasing cracks [27].

### 3.2. Full Saturation Probe

It is not possible to use standard eddy current probes to inspect ferrous or magnetic stainless steel tubes, such as Monel 400 (ferromagnetic copper-nickel alloy) because of the little or no penetration of eddy current fields at practical test frequencies due to their high permeability. Furthermore, variations in permeability of these tubes cause eddy current responses which are orders of magnitude greater than any defect indications. In order to detect the defects in these tubes, it is thus necessary to magnetically saturate the tube material using a strong static magnetic field and reduce the effective permeability of the tube. This increases the penetration depth and also reduces indications due to permeability variations.

Saturation eddy current probes are conventional eddy current probes with integrated strong permanent magnet bias to magnetize the tube material. Magnetic saturation to reduce the permeability ensures the adequate eddy current depth of penetration in order for the internal probe to detect defects that start from the outer diameter surface of the tube. These types of probe are used in the case of partially ferromagnetic materials, such as copper-nickel alloy and stainless steel [28]*.*

The problem with full saturation eddy current probe is the need for ensuring that ferromagnetic material is saturated. However, with a reference calibration tube, this can be verified.

### 3.3. Rotating Bobbin Probe

Bobbin probes are fast and effective in initial detection and sizing the degradation, but insensitive to circumferential cracks and defects around transition zones. Surface riding probes such as Rotating Pancake Coil (RPC) and Plus Point (+Point) are supplemental probes used in the examination of tube defects and other areas of concern, such as transition and U-bend areas [29,30].

The pancake and plus point coils are connected to motor units, pressed to the tube surface by springs and rotated by a motor circumferentially inside the tube in a helical pattern. These probes can detect both axial and circumferential cracks and provide information about defect morphology [30,31].

Rotating pancake coil probes typically contain 3 surface-riding pancake coils placed around the circumference. Plus point coils are two orthogonal coils connected in differential mode crossing at a point which is affected simultaneously by material and geometrical distortions such as lift-off and defects during the inspection.

In the commercial rotating probes, there are three coils placed spatially 120 degrees apart, including one plus point differential eddy current coils, and two high and low-frequency pancake coils. Each scans the inner surface of the tube in a helical path, thus a C-scan image is obtained from these probes. The three coils are excited at multiple frequencies, typically, 200, 300 and 400 kHz.

The rotating probe is sensitive to defects of all orientations and has high resolution and improved sensitivity to characterize and size defects. However, it is sensitive to probe lift-off. In order to minimize the effect of lift-off, the pancake plus point coils are spring loaded to contact with the tube wall inner surface. The mechanical rotation of the coils causes serious wears so the probe is prone to failure. In some cases such as CANDU reactor tubes, the situation is even worse, because there are internal magnetite deposits on tube wall which reduce the probe life significantly due to the wear. Furthermore, these magnetic deposits can also obscure the signals from defects. Since the probe has a helical scan pattern, the scan speed is slow, around 0.01524 m/s and 120~80 times slower than that of bobbin probes. Hence, the inspection time and cost increase significantly.

### 3.4. Array Probe

The array probe belongs to the excitation-receive type. The excitation and receive coils of array probe coils are magnetically coupled. The excitation (active primary) coils are driven by time-harmonic AC at several frequencies. Induced voltages in receive (passive secondary) coils are generated by the change of magnetic flux through the coil windings. Any defect in the material specimen that affects the flow of eddy current and the changes of magnetic flux through the windings of the receive coil is detected and characterized.

**Figure 6:**
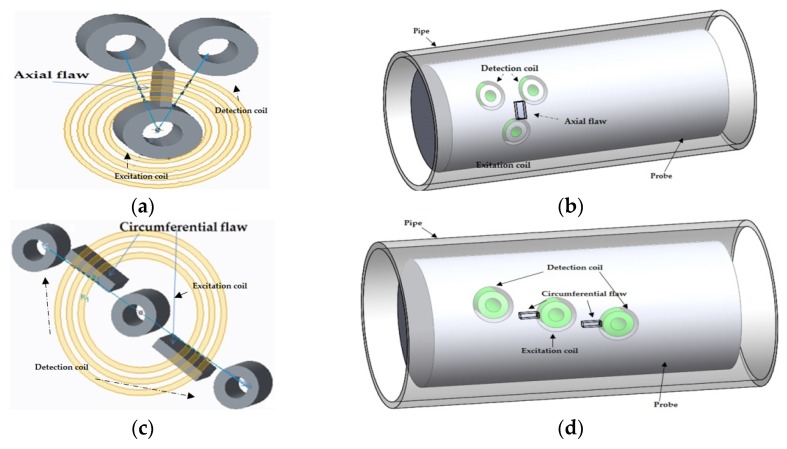
Axial and circumferential channels of array probe: (**a**) Axial channel configuration; (**b**) illustration of the axial channel excitation and detection coil location on the eddy current testing probe (**c**) circumferential channel configuration; (**d**) illustration of the circumferential channel excitation and detection coil location on the eddy current testing probe.

A typical array probe is composed of an array of pancake eddy current coils. These pancake coils form axial and circumference channels separately at different time slots. Figure 6 shows the axial and circumferential channels of an array probe. The axial defects disturb the eddy current induced by axial channel excitation coil and this is detected by this channel’s two detection coils. The circumferential defects cut the eddy current induced by the circumferential channel excitation coil and the indication of the defects are present in the corresponding detection coil.

### 3.5. C-Probe

**Figure 7:**
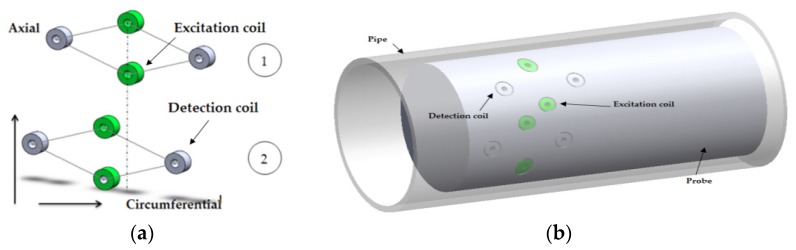
General setting for a C-3 probe: (**a**) Excitation and detection coil configuration; (**b**) illustration of the excitation and detection coil location on the eddy current testing probe for a C-probe.

The C-Probe C-3 was the first initial array probe built by Cecco in the 1990s, and later C-4, C-5 probes were produced [32,33]. Two circumferential arrays of excitation and detection pancake coils are arranged with a small degree of shift along the circumference to improve the resolution along the circumferential direction, as shown in Figure 7.

### 3.6. X-Probe

The X-Probe was built in the 1990s. There are a number of coils ranging from 8~19 in each row (depending on tube diameter). Special designs are also made for tight radius U-bends. Array probes are usually a combination of a X-probe and a bobbin probe on the same head, so the inspection times decrease greatly and re-visiting tubes with different probes are eliminated. It has however a complicated excitation and data acquisition system, which makes the probe costly [34].

**Figure 8:**
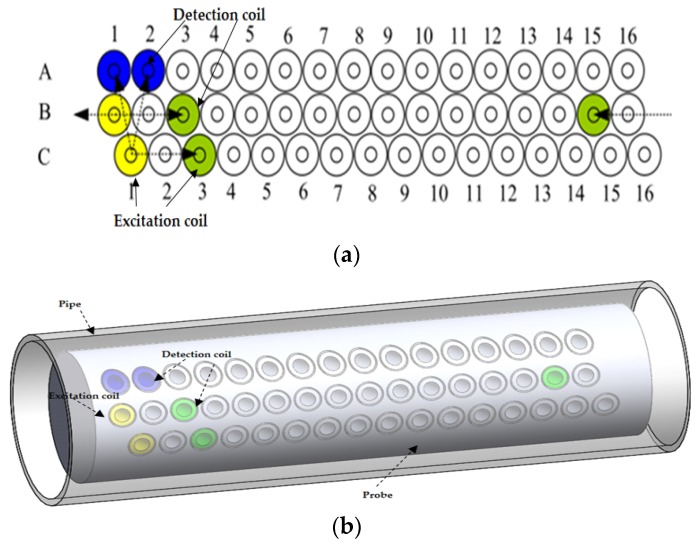
Axial and circumferential channels of array probes: (**a**) Excitation and detection coil configuration; (**b**) illustration of the excitation and detection coil location on the eddy current testing probe for an array probe.

An array of pancake coils covers 360 degrees of the circumference of inner pipe surface, as shown in Figure 8. There are three rows of pancake coils, with 16 in each row. Instead of rotating a single pancake probe circumferentially by a motor over the circumference as is done in RPC, multiplexing techniques are used to switch between axial and circumferential channels separately at different time slots around the circumference. The first two axial channels are obtained by transmitting from C1 to A1 and A2. The first two circumferential channels are obtained by transmitting from B1 to B3 and B15 and from C1 to C3. The excitation and detection coils are activated at different multiplexed times, enabling each detection coil to detect a signal from one excitation coil at a time. The other channels are generated in the same manner after incrementing the numbers by one, giving a total of 32 axial and three sets of 16 circumferential channels in this example (with a 22.5° angular step-size for circumferential channels and 11.25° step-size for axial channels). Note that each of the 32 axial channels covers 11.25° around the circumference of the tube, and each circumferential channel covers 22.5°. Data are collected as the probe is pulled along the axis of the pipe, giving circumferential and axial defect information at each axial and circumferential location in the tube [35,36]. Thus, the data collected are in the form of a 2D image. Since C01 works both on excitation and detection mode, thus the control circuit becomes much more complicated.

The array probe is capable of detecting and characterizing circumferentially and axially oriented defects such as cracks, as well as volumetric defects. It has 10 times faster inspection speed than RPC. Multiple uniformly spaced identical pancake coils ensure equal sensitivity over the circumference [8].

### 3.7. Smart Array Probe

The smart array probe is an improved version of the X-probe with the following characteristics that are different from the X-probe [37,38]:

- One transmitter and four receivers
- Every coil works in either excitation or detection mode
- Simple DAQ circuits
- Circumferential mode with higher resolutions
- No need for axial position correction
- Less time slots

**Figure 9:**
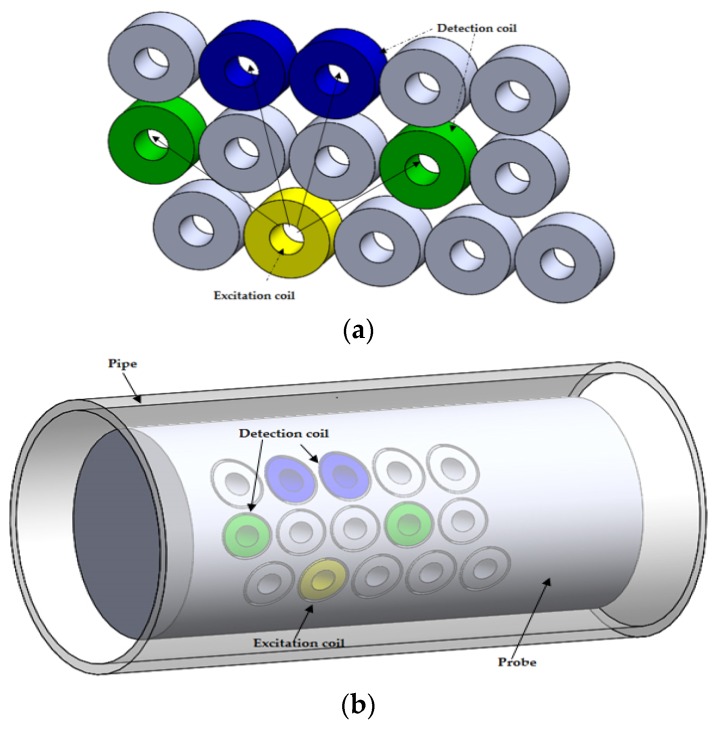
Smart array probe: (**a**) Excitation and detection coil configuration; (**b**) illustration of the excitation and detection coil location on the eddy current testing probe for a smart array probe.

As shown in Figure 9, there are only one excitation coil and four different detection coils. This new design highly simplifies the control and DAQ circuits and also reduces the time slots by half.

### 3.8. Intelligent Probe

The intelligent probe was built by Mitsubishi Heavy Industries and the first field trail took place in 1997. The excitation coils are specially designed as an inclined coil, which is effective in both axial and circumferential defects. The detection coil is a thin film pickup coil connected with a built-in electronic preamplifier circuit. The probe also combines a bobbin coil with array coils. It can detect all orientation defects in a single pass [39,40].

In summary, the array probes have high resolution, and relatively faster scanning speed compared with the rotating probes, however, they are costly and have complicated control circuit and signal post-processing schemes. If a specimen’s shape and size are changed then a new probe design is required.

### 3.9. Rotational Magnetic Flux Sensor

The rotating magnetic flux sensor was proposed by Enokizono in 1997. This sensor is of the excitation-detection type. Two pairs of pancake-type coils with iron cores are arranged orthogonally from each other and are excited by two phase alternating currents with a 90 degree phase shift. Thus a rotational magnetic flux with constant amplitude is generated in the specimen. Three axis searching coils measure the magnitude of the magnetic leakage flux density, which is perturbed by defects in the plate or tube. 

**Figure 10:**
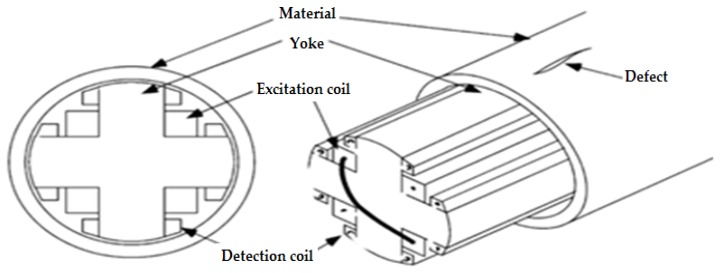
Rotating magnetic flux sensor for pipe and tube inspection.

Two type of probe designs are used for flat plate inspection, where iron cores are replaced by two ferromagnetic yokes. Two pairs of excitation coils and the search coils are both wound on the yoke as represent in the figure. A modified design of the probe renders it possible to inspect a tube with the same principle as shown in Figure 10. However, this type of sensor is only applicable for ferromagnetic materials, since it needs a return path for the magnetic flux [41,42].

### 3.10. Rotating Magnetic Field Probe

**Figure 11:**
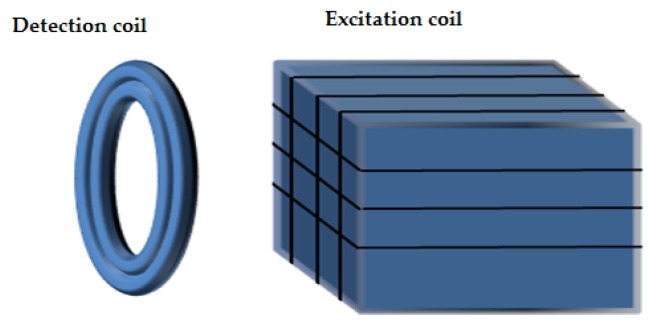
Two phase rotating field eddy current probe described by Birring [43].

A rotating field eddy current probe was built and tested by Birring in 1999 for use in small diameter, non-magnetic tubing. The probe is of the excitation-detection type with two orthogonally wound excitation coils and a pancake detection coil underneath, as shown in Figure 11. The excitation coils are excited by two sinusoidal currents with a 90 degree phase shift with each other, thus, a rotating magnetic field is created in the specimen under the excitation coils. The probe produces a bipolar response in the presence of cracks or notches. The phase angle of response signals is measured and used for estimating the depth of volumetric defects [43].

Three different frequencies are used for the excitation, such as 100, 210, and 300 kHz. The specimen is 0.0127 m outer defect tube with 0.0011938 m tube wall thickness. The tube material is Inconel Alloy 600. There are four electrical discharged machine (EDM) defects, which are 0.000254 m in width and 45 degrees in circumferential extent, with different depths varying between 40%–100%. The detection pancake coil is 0.0127 m in diameter. From the test results, the phase of the signals relates to the depth of the defect when 210 kHz is used as the excitation frequency.

**Figure 12:**
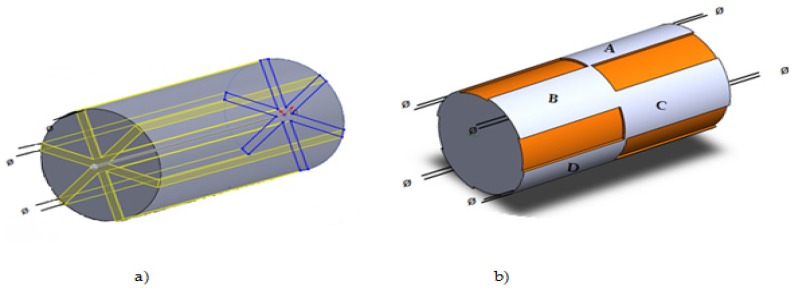
Inner rotating field eddy current transducer: (**a**) three phase rectangular coils; (**b**) A, B,C….,H: flat rectangular pickup coils.

An inner eddy current transducer with the rotating magnetic field is also proposed by Grimberg for tube inspection, as shown in Figure 12a,b. The excitation coils are three rectangular windings with the same turn numbers and placed 120 degrees spatially apart. These windings are then excited by three phase balanced sinusoidal alternating currents. Thus, a rotating magnetic field is generated by these three windings. The detection coils are eight flat coils placed on the external surface of the probe cylinder and connected to a multi-channel eddy current control equipment. For tube inspection the probe is pushed and pulled by a computer controlled system [44,45].

The probe can detect certain material discontinuities and provide information about the angular position of discontinuities. Sample defects such as outer defect circumferential grooves and other simulated corrosion on Inconel 800 tubes with outer 20 mm and thickness 1.8 mm are detected by the probe at an excitation frequency of 102 kHz.

In summary, rotating field eddy current probes were proposed to overcome the drawbacks of conventional rotating probes and array probes. The basic idea is to produce a rotating magnetic field electrically instead of by mechanical rotation of the probe. The probe generates a rotating magnetic field using three identical rectangular windings, and a detection coil is used to sense the response signals from defects. The probe is the excitation-detection type with high signal to noise ratio, and sensitive to defects of all orientations, which is functionally equivalent to a rotating probe coil (RPC). Furthermore, the probe has a higher scan speed than RPC and simpler control circuit than array probes. There are multiple excitation and detection coil configurations, according to different applications. The sensitivity and resolution are highly dependent on the detection coil. The probe can be either inside-tube probes or outer encircling probes, depending on the test sample.

## 4. The Influence of Various Parameters on the GMR Measurement

There are many parameters that affect the GMR measurement. The most important parameters will be discussed in this section.

### 4.1. Structural Quality of Giant Magnetoresistance Sensor

The quality surface layer of the GMR structure is an important factor for GMR sensor. Alpe *et al.* [46] have studied the relationship between the quality of the surface layer and the magnetic resistance properties of the GMR structure layer. They found that by heating the layer structure of Fe/Cr at 300 °C the magnetic resistance (MR) layer increases.

Petit [47] investigated the relationship between the formation of a rough surface by an annealing process at different temperatures. His research shows an annealing process for the structure of the GMR layer Fe/Cr at temperatures greater than 425 °C will reduce the magnetic resistance of the structure by 25% and the quality of the surface layer of the structure is increased. This change is due to a reduction in GMR antiferromagnetic coupling structural properties.

### 4.2. Thickness Structure Layers of Giant Magnetoresistance Sensor

**Figure 13:**
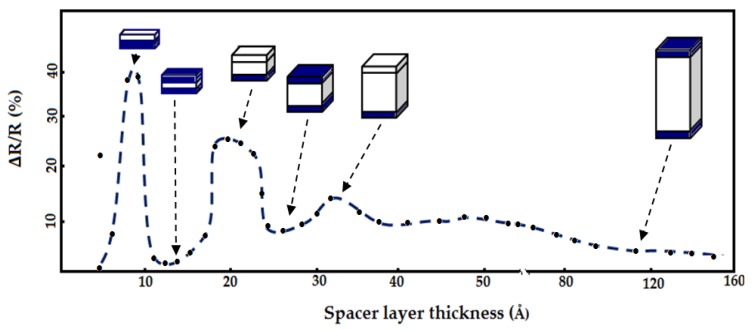
Magnetoresistance *versus* Cu spacer thickness for Co/Cu GMR multilayers at room temperature by Parkin *et al.* [48].

Parkin *et al*. [48] introduced in 1990 a sputtering technique in the fabrication process of GMR structures. With this technique, the GMR structure growth process is fast compared to the MBE process and this enabled them to study the effects of spacer thickness on the magnetic resistance of GMR structures. They proved there is coupling between the ferromagnetic and antiferromagnetic material in GMR structures of Fe/Cr. When the thickness of the spacer in the Fe/Cr GMR structure is increased, the magnetic resistance of the structure began to change from infinite to zero. They found that the magnetic resistance properties decrease when the thickness of the Cu spacer increased, as shown in Figure 13.

**Figure 14:**
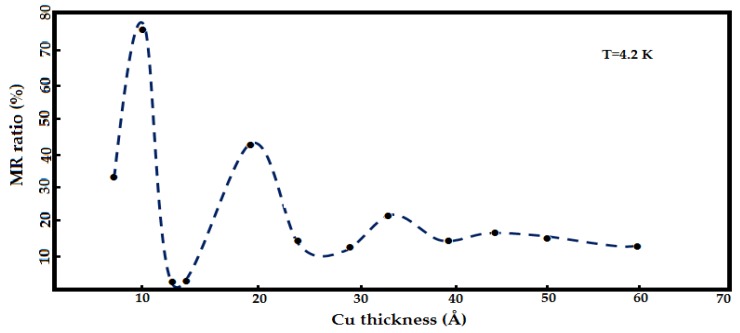
Variation of the MR ratio as a function of the Cu thickness by George *et al.* [49].

A study by George *et al*. [49] demonstrated that the high magnetic resistance on Co/Cu GMR structure layers is caused by the scattering effect of the electron spin in the surface layer of Co and Cu. They found that the effect of scattering occurs at the interface. They found that when the temperature of the Cu layer increased to 4.2 K, the magnetic resistance of the structure will be changed to a certain point as shown in Figure 14 before it becomes almost horizontal when the thickness of this layer is 50 Å.

As a conclusion, the GMR layer structure should be optimized as possible for high magnetic resistance properties. GMR layer structures should be thin, but not too thin to avoid a thick formation of pinholes.

### 4.3. Temperature

The effect of magnetic resistance decreases monotonically by a temperature factor between 1.5 to 3 at a temperature between 4 K and room temperature. The increase in temperature increases the number of electrons scattering in NM layers, causing the number of electrons moving between the layers of GMR structures to increase. This result reduces the efficiency of the GMR mechanism with increasing temperatures.

**Figure 15:**
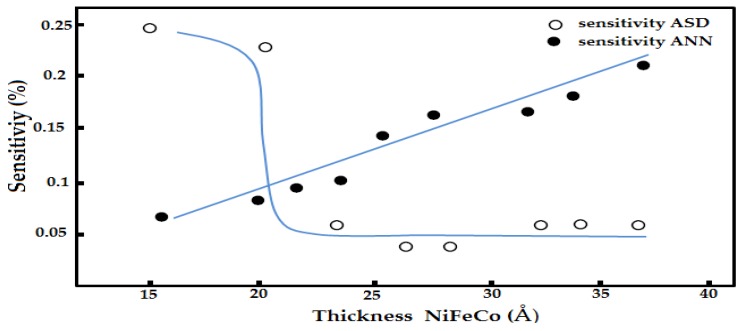
GMR sensitivity in as-deposited (ASD) and annealed (ANN) states as a function of the NiFeCo layer thickness by Hossain *et al.* [50].

In recent years, several studies have focused on the thermal stability of GMR structures and the relationship between temperature and magnetic resistance (MR) in GMR structures. To enhance the thermal stability of the GMR layer structure, Hossain *et al*. [50] proposed a GMR structure composed of NiFeCo/Cu layers. The layer structure is fabricated using a sputtering method and has a thick of the magnetic layer. Cu spacer thickness is set to 2.3 nm and a layer of magnetic material that is changed from 1.7 nm up to 3.7 nm. Their observations show the structure of the layer thickness of 3.7 nm NiFeCo shows good in thermal stability with high sensitivity, as shown in Figure 15.

**Figure 16:**
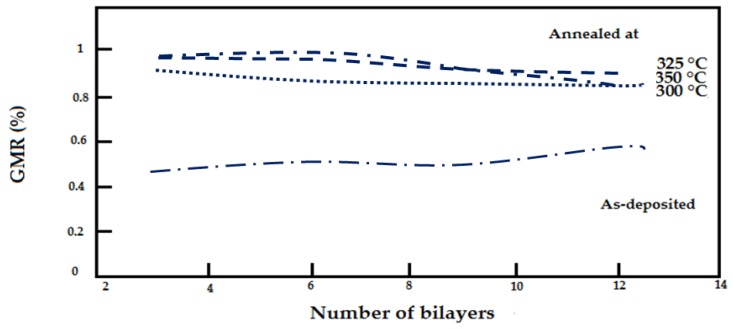
The annealed GMR multilayer in a vacuum at 300 °C, 325 °C and 350 °C. by Siritaratiwat *et al.* [51].

In a different study, A. iritaratiwat *at el*. [51] investigated the effect of the annealing process on the NiFe/Cu layer of a GMR structure. The thickness of the NiFe layer is 2.5 nm and the Cu spacer is 5 nm. They heat the GMR structure for 2.5 h at 300 °C, 325 °C and 350 °C in a vacuum chamber. In a second experiment, they heat the GMR structure with same experimental parameter settings but with a stream medium of argon gas. The results are shown in Figure 16.

**Figure 17:**
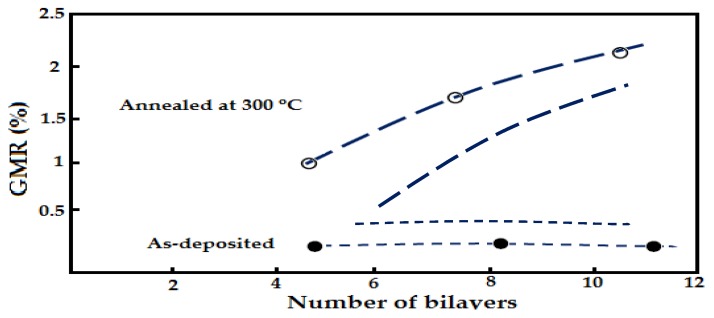
Annealed GMR multilayer in flowing argon by Siritaratiwat *et al.* [51].

As shown in Figure 17, when a GMR structure is heated at a temperature (300 °C, 325 °C and 350 °C) under vacuum conditions there is an improvement in the structure of the magnetic resistance up to 1%, while the GMR heated in flowing argon medium has improved magnetic resistance MR structure properties by up to 2.5%.

## 5. Factors Affecting the Eddy Current Testing Inspection

Many factors, other than defects and cracks, will influence the eddy current inspection. The signal from an eddy current probe is a compilation of responses, including responses from flaws and defects, sample geometry, and probe lift-off [52,53,54]. Therefore, it might be hard to isolate a single effect. Successful evaluation of flaws or any other surface properties is possible when the other factors are known. The main factors affecting the coil response are:

### 5.1. Exciting Coil Frequency and skin Depth Effect

The capabilities of conventional eddy current testing using a single frequency current coil excitation are limited to detecting defect depth in one or two skin depths. Multi-frequency excitation coil eddy current testing was introduced to obtain additional different depth defect profile information. Sine wave current excitation coils with frequencies between 100 Hz up to a few MHz are used to check profile material defects in different layers [55,56].

The exciting coil frequency is a very important factor in determining the depth of the located defect. For subsurface defect inspection, a low frequency will be applied [57]. On the other hand, for detecting surface defects, a high frequency needs to be applied. Table 2 summarizes the values of skin depth for several materials at different frequencies.

Eddy current density in metallic materials decreases exponentially in proportion to the depth of the material. The standard penetration of eddy currents is where the strength of the eddy current is 37% of the strength of the eddy current on the surface of the material [58]. Apart from the frequency current coil, the penetration depth of eddy current depends on the the electrical conductivity and permeability properties of the material tested. Depth penetration of the eddy current in the material can be calculated using the following formula [59,60]:

(1) $$\delta =\frac{1}{\sqrt{\pi f\mu \sigma }}$$

where δ: Skin depth (mm); f: Excitation frequency of the coil; µ: conducting material permeability and σ: conducting material conductivity. As the magnitude of the eddy current decreases exponentially with the depth of the tested material, the amplitude of the magnetic field along the X-axis generated by the eddy current in the material under test can be represented by the following formula [61,62]:

(2) $$J\left(x\right)=J_{s}Xe^{-x/\delta }$$

**Figure 18:**
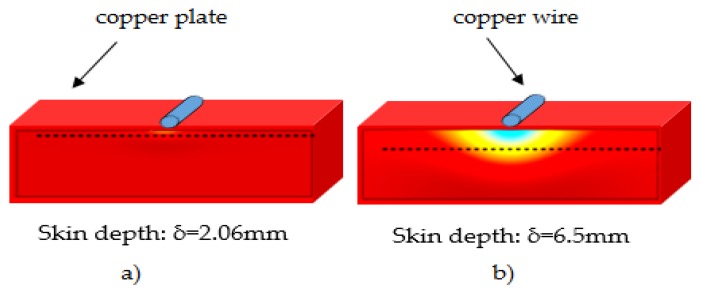
Skin depth effect in eddy current testing for copper: (**a**) 100 Hz exciting coil frequency; (**b**) 1 kHz exciting coil frequency [63].

Based on Equation (2), the depth of eddy current penetration into the conductor material also depends on the coil current frequency. The low frequency of excitation coil current should be used for deep subsurface defect inspection. Figure 18 shows the simulation of skin depth effect using different frequency current excitation coils. 

The depth penetration of eddy current in the material is measured as a skin depth of the material. Table 2 shows the skin depth with different frequencies for metals. The inclusion of a broad range of frequencies led to the pulsed excitation in ECT technique that applies a square, triangular, or a sawtooth waveform as a source current [65]. PEC techniques measure transient signals that contain a broad spectrum of frequencies and provides capabilities to detect and characterize deep corrosion and hidden defects. This technique also offers advantages in correlating depth information with time-dependent characteristics in the response signals [66,67].

### 5.2. Material Magnetic Permeability

By using a metal that has low permeability, this causes the eddy current to penetrate deeper and *vice versa*. In addition to these factors, there are some other factors related to the geometry of the sample under test and the shape of the defects (line, point, corner crack..., *etc*.). The coil shape depends on the geometry of the sample and the excitation frequency depends on the thickness of the sample.

### 5.3. Lift-off

**Figure 19:**
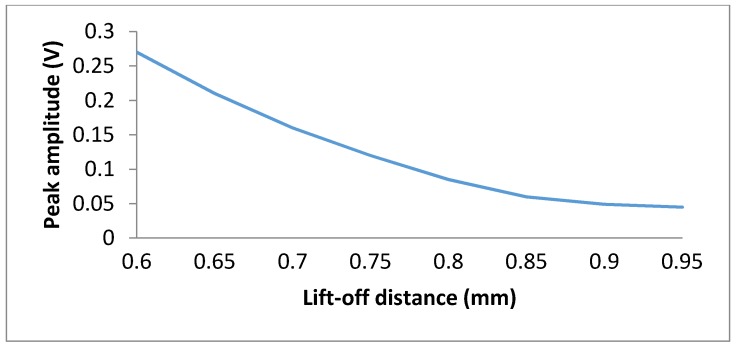
A peak amplitude as a function of lift-off distance between probe and specimen surface.

Lift-off is the distance between the probe eddy current testing (exciting coil and receiving sensor) and the surface of the conducting specimen under test. When lift-off increases, the secondary magnetic field on the specimen surface decreases, which causes reduced sensitivity of the probe. Dogaru *et al.* [68] investigated the lift-off as a function of the GMR sensor amplitude output signal. Figure 19 show the peak amplitude of an output voltage GMR sensor changes with the lift-off distance.

### 5.4. Conductivity of Material

When the conductivity changes, the magnetic field changes too, and this affects the output of the receiving magnetic sensor on the probe. The conductivity of metals is affected by heat treatment, age hardening temperature, chemical deposition and residual stresses. The conductivity is measured by referring to the International Annealed Copper Standard (IACS). Table 3 summarizes the conductivity and resistivity of selected conductive materials (Moulder *et al.* [69]).

### 5.5. Limitations of Coil Sensor in Eddy Current Probe

Traditional EC methods use coils as sensors (pick-up coils) to measure changes in the magnetic field. Based on Faraday’s law of induction, the voltage response of a pick-up coil is proportional to the rate of change of the induced magnetic field not the magnetic field itself. Therefore, it results in poor SNR ratio particularly at low frequencies [66,70]:

(3) $$V_{coil\;signal}=N\pi r^{2}\frac{dB}{dt}\propto N\pi r^{2}f$$

where *N* is the number turns of coil wire, $\pi r^{2}$ is the area of the loops and *dB/dt* is the rate of change of magnetic field that is proportional to the operating frequency *f*.

**Figure 20:**
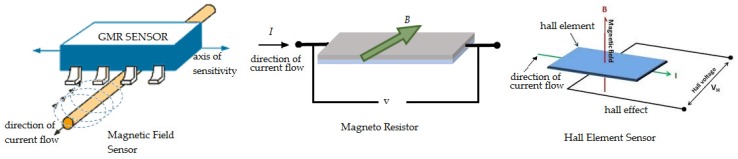
Hybrid probe [26,72]: ECT coil with magnetic field sensor.

Consequently, coil sensors are fundamentally limited by their poor sensitivity at low frequencies. Unfortunately, sensitivity at low frequency is needed in the inspection of thick components and subsurface flaws. Similar to traditional coils, planar coils also present limited sensitivity when they are used as pick-up or inductive coils for sensing low-frequency magnetic fields [71]. Alternately, ECT probes that are operated in hybrid mode have been developed to overcome those limitations. As shown in Figure 20, hybrid ECT techniques employ conventional or planar coils to generate eddy currents and utilize magnetic field sensors to directly measure field variations associated with discontinuities [26,72].

## 6. Compensation Techniques in Eddy Current Testing Probes

Many factors affect the accuracy of defect measurement using the eddy current testing technique. Factors such as temperature, hysteresis, edge effect and lift-off should be considered for accurate inspection. Several researchers have focused on solving and compensating the factors that affect the eddy current measurement. Based on the 3D Helmholtz coil, Jixi *et al*. [73] have developed a special system for testing characteristics of a GMR sensor. They used a series resonance circuit to compensate the increase of inductive impedance when the frequency of the exciting coil increases. Experiments showed the precision of GMR sensor measurement increased if the density of the magnetic field was in the linear measurement range. However, the error is still high with a percentage of 20% due to hysteresis effects on the GMR sensor output signal. Pelkner *et al.* [74] analyzed the optimum configuration of a GMR sensor array for optimum defect inspection. They analyzed the magnetic flux distribution (MFL) model to represent multi-sensor function parameters. Their test results showed that a sensor array arranged in a configuration of half bridges and Wheatstone bridges can minimize the effect of temperature on GMR sensor array measurements.

Lift-off is the main factor in ECT signal reading errors. The lift-off may be described as the distance between the probe and the test piece. Its variation adversely affects the ECT inspection in many applications [75] so it is therefore considered as the main noise factor in ECT signal analysis. To avoid lift effect the probe distance with the test piece should be maintained, but in a real application, it is difficult because of factors such as irregular test surfaces, varying coating/lagging thicknesses and operator movement [76].

To compensate lift-off effect, Yin *et al.* [75] published a research finding on an analytical model based on multi-frequency excitation and coil design aimed at the reduction of this effect. Their findings showed that the phase spectra of such coil designs are essential to remove the lift-off invariant. Xu *et al.* optimized an ECT coil design in an attempt to reduce lift-off effects [77]. In a different study Lopez *et al.* [78] proposed a wavelets technique to remove noise from probe wobble in steam generator tube inspection. A normalization technique has been proposed by Tian *et al.* [79] to minimize lift-off effects. They demonstrated it could be used in metal thickness measurement under non-conductive coatings and for microstructure analysis where the output signal is affected by the presence of lift-off effects. In another study, Fan *et al.* [80] has presented a lift-off analytical model for probe wobble in heat exchanger tube inspection. Another way of dealing with this effect is by using invariant point features called lift-off point of intersections, which has been successfully used to estimate the conductivity of test materials in [76] and for corrosion mapping in gas pipelines [81].

When an eddy current testing probe is at the end of a specimen, a edge effect phenomenon sets in. At the edge of test piece the eddy current in the test piece is distorted as the current cannot flow. In order to avoid it being mistaken for a defect signal, Yang *et al.* [82] suggested small probes should be used for defect inspection near edges. They developed a post-processing subtraction algorithm to compensate for the edge signal effect. In contrast, Theodoulidis *et al.* [83,84] proposed a mathematical model to calculate the field of a coil probe on the edge of a conductive material. This model elicited some analytical field formulations that gave better insight into this phenomenon and could form the basis of a process for solving edge effect-associated challenges. Table 4 provides a summary of compensation techniques used in eddy current testing probes.

## 7. Application of GMR Sensors in Hybrid Eddy Current Testing Probes

Implementation of the GMR sensor as a magnetic receiver in eddy current testing probes has shown significant improvement in term of efficiency in defect inspection. Conventional eddy current probes using a coil as a magnetic detection cause the noise ratio of the defect signal to be high and this causes the accuracy of defect interpretation to be inaccurate. GMR magnetic sensors directly measure the magnetic field changes and this increases the sensitivity of the probe in detecting a subsurface defect that is far below the tested surface material. For this reason, the study of the application of GMR sensors in non-destructive testing remains the focus of researchers.

Postolache *et al.* [58] developed an eddy current testing system using GMR sensors to detect and measure the size of defects on aluminum plates. They used various different frequency excitation systems and processing methods, and a neural network to classify the size of the detected defects. Initial tests showed their system can detect and classify types of defects within a limited range of defects. This system can be improved by optimizing the detection and classification system. Jedlicska and Weigel [95] introduced a method for increasing the GMR sensor measurement accuracy by eliminating hysteresis effects in GMR sensor output signals. A mathematical model of hysteresis was implemented in an eddy current testing system based on a GMR sensor. The system is fully controlled by LABVIEW software. Comparison of simulation results with experimental results showed significant results. However, these systems do not consider other factors in eddy current testing such as material permeability properties and temperature effects on measurement. Meanwhile, Bernieri and Betta [96] introduced measurement and calibration procedures for GMR sensors to reduce the effect of hysteresis and nonlinearity. They introduces procedures to improve the accuracy of the reading in DC and AC excitation magnetic fields up to 98.2% and 99.4%.

In different studies, Winncy *et al.* [97] have designed a robot for internal pipe defect inspection. The sensor array consists of four GMR sensors which have high sensitivity (0.9 to 1.3 mg/V). The sensor housing is made using a polycarbonate material to minimize the disturbance on the magnetic field. Four GMR sensors are arranged at a uniform distance and a PIC microcontroller is used as the main controller and data processing system. Permanent magnets are used to generate magnetic fields. A Fast Fourier transform is used to differentiate the defect signals. Their experiments showed that different types of defect generate different harmonic signals. However, the use of permanent magnets to generate the magnetic field limits their depth of defect detection. Yang *et al.* [98] used a GMR sensor to investigate the quality of aeronautical structures. The focus of their study was the defects under the riveted headings used to connect the airplane wing structure. The exciting rotating magnetic field is used to enable the GMR sensor to show a uniform sensitivity. Mathematical modeling and experimental validation were conducted to show how the different defects give different signals. However, this study could be improved by using an array of GMR sensors to obtain clear structure defects. Meanwhile, Gao *et al.* [99] used the amplitude-phase of the GMR sensor output signal to classify the type of defect. Their experimental results show that defect classification is more accurate using the analysis of the phase signal of the GMR sensor output.

GMR sensors have been used in eddy current testing to measure the depth of cracks in the structure of airplanes by Pasadas *et al.* [100]. A high-density magnetic field is applied to the entire cracked structure. Features of 2-D defects were successfully obtained with accurate dimension of defect information. Chao *et al.* [101] have been integrating GMR sensors with Field Programmable Gate Arrays (FPGAs) for the development of eddy current nondestructive testing systems which can detect cracks up to 10 mm wide. Through a series of experiments, they proved the characteristics of the phase signal output of the GMR sensor can provide more accurate defect information compared to amplitude signal analysis.

For detailed defect profiling, five GMR sensors were integrated into a eddy current testing system by Postolache *et al.* [58]. Excitation of the uniform magnetic field is generated by using alternating current and direct current sources. The system works automatically, which can give a defect in the form of a 2-D image. Meanwhile Munoz *et al.* [102] constructed their eddy current probe using a GMR sensor and a permanent magnet for exciting a magnetic field. They studied the relationship between the orientation of defects with the sensor GMR output signal. Iron plates with two-dimensional defect depths of 0.5, 1.0, 1.5, 2.0, 2.5 and 3.0 mm and a width of 0.25 and 0.5 mm were fabricated to test the precision of the probe. The experiments showed the signal output of GMR is sensitive to the orientation of defects. Table 5 summarizes previous studies that applied GMR sensors in non-destructive eddy current testing.

## 8. Conclusions

The eddy current testing technique has played an important role as one of the main non-destructive techniques chosen by industry. The reliability of the ECT technique in an inspection of the material quality was proven to show better capability in defect detection. Although it shows great advantages compared to other non-destructive methods, the eddy current technique has a particular drawback that originates from its underlying principles, however the implementation of GMR sensors in eddy current testing probe designs overcomes the weakness and limitations of non-destructive eddy current testing inspection. Implementation of GMR sensors in ECT probe design significantly increases the measurement accuracy and scope of the inspections possible using ECT techniques.

Lift-off and edge effects are the main factors affecting the ECT signal causing erroneous data interpretation. The evolution of simulation software and the intelligent algorithms has led to various analytical solutions that can thus compensate the unwanted signals, promising a more comprehensive defect profile analysis and unambiguous resolution of deep hidden defects in thick structures.

## Figures and Tables

**Table 1:** Major NDT Methods—A comprehensive overview [7].

Method	Principles	Application	Advantages	Limitation
Visual Testing	Uses reflected or transmitted light from test object that is image with the human eye or other light sensing device	Many application in many industries ranging from raw material to finished products and in-service inspection	Can be inexpensive and simple with minimal training required. Broad scope of uses and benefits	Only surface conditions can be evaluated. Effective source of illumination required. Access necessary
Penetrant Testing	A liquid containing visible or fluorescent dye is applied to surface and enters discontinuities by capillary action	Virtually any solid non-absorbent material having uncoated surfaces that are not contaminated	Relatively easy and materials are inexpensive. Extremely sensitive, very versatile. Minimal training	Discontinuities open to the surface only. Surface condition must be relatively smooth and free of contaminants
Magnetic Particle Testing	Test part is magnetized and fine ferromagnetic particle applied to surface, aligning at discontinuity	All ferromagnetic materials, for surface and slightly subsurface discontinuities; large and small parts	Relatively easy to use. Equipment/material usually inexpensive. Highly sensitive and fast compare to PT	Only surface and a few subsurface discontinuities can be detected. Ferromagnetic materials only
Radiographic Testing	Radiographic film is exposed when radiation passes through the test object. Discontinuities affect exposure	Most materials, shape, and structure. Examples include welds, castings, composites, *etc.*.. As manufactured or in service	Provides a permanent record and high sensitivity. Most widely used and accepted volumetric examination	Limited thickness based on material. Density, orientation of planar discontinuities is critical. Radiation hazard
Ultrasonic Testing	High frequency sound pulses from a transducer propagate through the test material, reflecting at interfaces	Most materials can be examine if sound transmission and surface finish are good and shape is not complex	Provide precise, high sensitivity results quickly. Thickness information, depth and type of flaw can be obtained from one side of component	No permanent record (usually). Material attenuation, surface finish and contour. Required couplant

**Table 2:** Typical Depths of penetration [64].

Metal	%IACS	Resistivity Ω·m	Permeability	36.8% Depth of Penetration					
				1 kHz	4 kHz	16 kHz	64 kHz	256 kHz	1 MHz
copper	100	1.7	1	0.082	0.041	0.021	0.010	0.005	0.0026
6061 T-6	42	4.1	1	0.126	0.063	0.032	0.016	0.008	0.004
7075 T-6	32	5.3	1	0.144	0.072	0.036	0.018	0.009	0.0046
Magnesium	37	4.6	1	0.134	0.067	0.034	0.017	0.008	0.0042
Lead	7.8	22	1	0.292	0.146	0.073	0.37	0.018	0.0092
Uranium	6.0	29	1	0.334	0.167	0.084	0.042	0.021	0.0106
Zirconium	3.4	70	1.02	0.516	0.258	0.129	0.065	0.032	0.0164
Steel	2.9	60	750	0.019	0.0095	0.0048	0.0024	0.0012	0.0006
Cast steel	10.7	16	175	0.018	0.0089	0.0044	0.0022	0.0011	0.0006

**Table 3:** Conductivity and resistivity of conductive materials.

Material	Conductivity (% IACS)	Resistivity (µΩ/cm)
Aluminum bronze	14.00	12.32
Aluminum 7075-T6	32.00	5.39
Aluminum 2024-T4	30.00	5.20
Aluminum 6061	42.00	4.10
Brass	28.00	6.20
Copper nickel 70–30	4.60	37.48
Copper	100.00	1.72
Gold	70.00	2.46
Monel	3.60	47.89
Copper nickel 90–10	9.10	18.95
Cast Steel	10.70	16.02
Hastelloy-X	1.50	115.00
Inconel 600	1.72	100.00
Lead	8.35	20.65
Magnesium	38.60	4.45
Phosphor bronze	11.00	16.00
Silver	105.00	1.64
Stainless Steel 316	2.33	74.00
Stainless Steel 304	2.39	72.00
Sodium	41.50	4.20
Ti-6AI-4V	1.00	172.00
Titanium-2	3.55	48.56
Tungsten	30.51	5.65
Zirconium	4.30	40.00
Zircalloy-2	2.40	72.00

**Table 4:** Compensation techniques used in eddy current testing.

Ref.	Research Area	Compensation Techniques
[85]	To remove the lift-off effect in PEC ferromagnetic material test piece inspection	Relative magnetic flux changing rate
[86]	Presented a simple model for metal thickness measurement that unaffected by lift-off effect.	Signal analysis base on multi-frequency phase signature
[87]	Developed ECT system based on three coils exciting coils to measure the plate thickness	Data analysis using peak frequencies of the sensor signal to estimate the thickness of the plate
[88]	Proposed a method for suppressing of lift-off effect in SMFM system	Signal deconvolution
[89]	Proposed ECT system with rectangular sensor configuration and time domain analysis and frequency domain analysis for defect classification.	Time domain analysis and frequency domain analysis
[90]	Proposed a method to reduce the lift-off effect in PEC deep defect measurement	Measure the defect dimension base on slope of the linear curve of the peak value difference sensor signal
[91]	Investigate the lift-off effect in the normalized impedance plane	Hough transform
[92]	Developed PECT system for ferromagnetic material electrical conductivity measurement	Mathematical model
[89]	Investigated the feature extraction techniques for PEC defect classification	Signal differential analysis
[93]	Developed ECT system to measure the thickness of nickel layer	3-D edge-based hexahedral nonlinear FEM
[79]	Investigated the effect of lift –off in PEC non-destructive testing	Normalization and two-stage operative process
[94]	Construct a system to measure the thickness of metal plates	Lift-off points of interception

**Table 5:** Summary of previous studies on application of GMR sensor in eddy current testing.

Author	Reseach Area	Signal Analysis Tool/Software Simulation	Observations
[103]	Defect classification in aluminium plate test pieces	Neural Network Processing	Probe optimization and defect classification using limited defect features.
[95]	To increase the accuracy of the GMR sensor by numerically compensating the hysteresis effect	Finite Impulse Response	Strongly reduced the hysteresis and optimized the probe design by increasing the speed of inspection
[104]	Optimize the eddy current testing probe for subsurface tiny crack defect inspection	Maxwell design simulation	The system is able to detect tiny defect cracks of up to 3 mm under the surface. Experimental results prove the main source of noise is the current excitation frequency.
[97]	Developed an eddy current testing probe based on an array of GMR sensors for pipe inspection	Fast Fourier Transformation	The array of GMR sensors is able to detect various types of defect. The signal output of the array sensor can be used to classify and define the properties of different defects.
[105]	Designed and construct an automatic eddy current system for inspection of an artificial straight defect in an aluminium plate.	Neural network/multilayer perceptron/competitive neural network/finite element simulation	Implementation of the neural network classification technique increases the accuracy of defect classification
[106]	Designed and developed an eddy current testing probe using a rotating exciting magnetic field for detection of radial cracks around a fastener	Finite element model simulation	The eddy current testing probe current shift exciting magnetic field is 90° in phase. The simulation and experimental results show the system is able to detect all orientations of a defect under the fastener
[107]	Developed an ECT system to classify multiple classes of defect thickness in conductive plates.	Support vector machine—SVM	The system successfully classified the thickness defect with an error lower than 1.52%.
[99]	Investigated the defect properties based on the phase signal of a GMR sensor	Finite element method (FEM) program	The experimental results proved the phase signal output of the GMR sensor provides more defect information.
[108]	Developed an ECT probe for surface defect inspection.	-	The probe was able to detect and measure an artificial defect with a dimension of 0.15 mm width and 0.2 mm depth.
[100]	Investigated the efficiency of defect detection using a differential pick-up coil and GMR sensor	-	Both sensors were able to detect defects with thicknesses of more than 1 mm. The GMR sensor detects the defect when the sensing direction crosses the edge defect while the pick-up coil needs the whole magnetic field to cross the defect to detect it.
[109]	Designed a 2-D magnetic field camera system to measure the properties of the magnetic field around inner and outer defects in a piping system	-	The system is able to sense the magnetic field in the radial and axial direction.
[110]	Proposed a method for deep subsurface defect inspection.	Finite Element Method (FEM)	Experimental and simulation show the system is able to detect deep subsurface defects
[111]	Investigated defect signals of an artificial rectangular straight defect in aluminium plates.	-	The experiments showed the direction of the defect is easy to detect if the defect is crossing the magnetic field.
[101]	Designed an ECT system based on a GMR sensor and a Field Programmable Gate Array (FPGA) as controller	Fourier Transform analysis	The system able to display the defect signals in amplitude and phase mode. A signal demodulation function has been realized for defect characteristic analysis.
[96]	Developed a low-cost ECT system with an automatic calibration system to reduce the uncertainty of GMR sensor measurement.	Static (DC) and dynamic (AC) analysis	The system is capable of inspecting defects using DC and AC exciting magnetic fields with a high percentage of accuracy
[112]	Developed an ECT system based on a GMR sensor for surface defect inspection	Polynomial regression	The system scans the defect in the direction of the sensor sensitive scanning area for accurate measurement.
[58]	Designed and optimized an ECT probe based on two planar excitation coils and a rectangular magnetic field biasing architecture	LabVIEW/sum squared difference (SSD) and normalized cross correlation (NCC)	Improved the inspection capabilities of the ECT probe with fast scanning time
[102]	Developed an ECT probe with radial magnetization	-	The 50° angle axis sensitivity of the GMR sensor to the defect orientation reduces by 28% the average value of the VD parameter
[113]	Investigated the performance of magnetic detection in an ECT probe for non-destructive inspection.	Numerical simulations	The results show a GMR sensor is better compared to the coil detector in term of sensitivity and dimensions.
[114]	Designed and modeled a magnetic field based on guide magnetic slopes	Finite element method (FEM)	The experimental results show the GMR sensor is sensitive only to the z-component of the magnetic field.
[94]	Implementation of an ECT to measure the thickness of metallic plates	-	The experiments show a frequency of 250 Hz is the optimum excitation coil frequency for maximum depth magnetic field penetration in the metal plate
[115]	Developed an ECT inspection systems using a GMR sensor	-	The system has the ability to inspect subsurface cracks at frequencies lower than 3.3 kHz
[74]	Investigated the optimal arrangement of a GMR sensor for optimum defect inspection.	Analytical model	The analysis shows the length and height of the GMR sensor influence signal strength loss by up to 10% a in 250 µm defect baseline
[116]	Investigated the characteristics of the current around an artificial crack for defect geometry identification	Fast Fourier transform/Tikhonov regularization algorithm	Characteristics of the current show a significant pattern with different geometry of cracks.
[98]	Proposed a novel invariance analysis for ECT signals in deep subsurface defects under fastener heads	Finite Element (FE)	Presented a reliable ECT inspection technique for different sizes and geometries of cracks under fastener heads.
[117]	Proposed an ECT system for inspection of hidden corrosion defects.	FEM	The inspection results show high accuracy with mean errors of less than 2%
[118]	Developed a PEC–GMR system for ECT non-destructive testing	Principal component analysis and the k-means algorithm	The system is capable of detecting cracks with a size of 1 mm located up to 10 mm subsurface
[119]	Developed an ECT–NDT system based on (GMR) sensors for circuit board (PCB) inspection	COMSOL Multiphysics	The system is capable of detecting and characterizing the type of defect track narrowing, circular holes and track dilatation.
[120]	Developed a general procedure for ECT defect sizing and classification in multilayered structures	Partial least squares (PLS)/kernel partial least squares (KPLS)	The KPLS regression method gives a better prediction performance compared to the PLS regression method
[121]	Proposed a novel ECT technique based on the induced velocity of eddy currents	Numerical model	The proposed method increases the sensitivity and the depth defect detection of the system.
[122]	Investigated the optimum asymmetrical coil-GMR configuration for surface defect inspection	-	The experimental results demonstrated that the intermediate peak does not have any influence on DV value with the depth of defects
[123]	Analyzed the sensitivity of GMR sensors and GMI sensors in detecting the magnetic field	Finite Element/Moments analysis	The experimental and modeling results show the GMR and GMI sensors are able to detect the changes of orientation of a magnetic field excited by using AC and DC current sources
[54]	Investigate the effect of lift-off in metallic plate thickness measurement	Linear Transformer Model/experimental	The lift-off, material conductivity and the plate thickness have a significant influence on the measurement of metallic plate thickness
[124]	Developed an ECT system based on a GMR sensor array for outer steel rope track defect inspection.	Finite element model	The experimental results reveal that the ECT system is able to detect both of LF and LMA type defects in the rope track.
[55]	Proposed a novel design of a rotating magnetic field ECT for SG tubes.	Finite Element modeling	The simulation and experimental results show that the probe is sensitive to defects in ferromagnetic and non-ferromagnetic tubes.
[125]	Investigated the performance of the PEC technique in material thickness measurement	Experimental	The method was verified experimentally to be suitable for material thickness measurement since the PEC method has deep magnetic penetration.
[126]	Enhanced the sensitivity of the ECT-GMR system using analysis of two signal GMR sensors	(3-D) Finite Element Mesh	Simulation results show that the proposed method improved significantly the sensitivity of the system in detection of multilayer subsurface defects
[127]	Developed an ECT-GMR system for inspection of defects under fasteners in airframe structures	Time domain and frequency domain features	Experimental results demonstrate the feasibility of the proposed approach for the detection of simulated cracks (less than 1 mm length) that are buried 4 mm deep in the second layer

## Abbreviations

The following abbreviations are used in this manuscript:

NDT: Non-destructive testing
ECT: Eddy current testing
GMR: Giant magnetoresistance
MR: Magnetoresistance
AFM: Antiferromagnetic
FM: Ferromagnetic
RPC: Rotating pancake coil
DAQ: Data acquisition,
NM: Non-magnetic
FEM: Finite element method
AC: Alternating current
DC: Direct current
